# Author Correction: Hypoxic gene expression in chronic hepatitis B virus infected patients is not observed in state-of-the-art in vitro and mouse infection models

**DOI:** 10.1038/s41598-020-75963-0

**Published:** 2020-11-03

**Authors:** Peter Jianrui Liu, James M. Harris, Emanuele Marchi, Valentina D’Arienzo, Thomas Michler, Peter A. C. Wing, Andrea Magri, Ana Maria Ortega-Prieto, Maarten van de Klundert, Jochen Wettengel, David Durantel, Marcus Dorner, Paul Klenerman, Ulrike Protzer, Efstathios S. Giotis, Jane A. McKeating

**Affiliations:** 1 Nufeld Department of Medicine Research Building, University of Oxford, Oxford, OX3 7LF UK; 2 Medawar Building, University of Oxford, South Parks Road, Oxford, OX1 3SY UK; 3 Institute of Virology, Technical University of Munich/Helmholtz Zentrum München, Trogerstrasse 30, 81675 Munich, Germany; 4 Section of Molecular Virology, Department of Infectious Diseases, Imperial College London, London, W2 1PG UK; 5 Cancer Research Center of Lyon (CRCL), INSERM U1052, and University of Lyon (UCBL1), Lyon, France; 6 School of Life Sciences, University of Essex, Colchester, C04 3SQ UK

Correction to: *Scientific Reports* 10.1038/s41598-020-70865-7, published online 24 August 2020

The original version of this Article contained a typographical error in the spelling of the author Ana Maria Ortega-Prieto, which was incorrectly given as Anna Maria Ortega-Prieto. This has now been corrected in the PDF and HTML versions of the Article.

