# Supplementary material for: Hypoxic gene expression in chronic hepatitis B virus infected patients is not observed in state-of-the-art in vitro and mouse infection models
Source: Sci Rep. 2020 Aug 24;10:14101. doi: 10.1038/s41598-020-70865-7 (PMC7445281; doi:10.1038/s41598-020-70865-7)
Supplement: Supplementary file 1 — Supplementary Information. [file 41598_2020_70865_MOESM1_ESM.pdf]

Hypoxic gene expression in chronic hepatitis B virus infected patients is not observed in state-of-art *in vitro* and mouse infection models.

PJ Liu[1], JM Harris[1*], E Marchi[2], V D'Arienzo[1], T Michler[3], PAC Wing[1], A Magri[1], AM Ortega-Prieto[4], M van de Klundert[3], J Wettengel[3], D Durantel[5], M Dorner[4†], P. Klenerman[2], U Protzer[3], ES Giotis[4,6] and JA McKeating[1**]

1. Nuffield Department of Medicine Research Building, University of Oxford, Oxford, OX3 7LF. UK.
2. Medawar Building, University of Oxford, South Parks Road, Oxford OX1 3SY. UK.
3. Institute of Virology, Technical University of Munich/Helmholtz Zentrum München, Trogerstrasse 30, 81675, Munich, Germany.
4. Section of Molecular Virology, Department of Infectious Diseases, Imperial College London, London, W2 1PG, UK.
5. Cancer Research Center of Lyon (CRCL), INSERM U1052, and University of Lyon (UCBL1), Lyon, France.
6. School of Life Sciences, University of Essex, Colchester, C04 3SQ, UK

** Shared first authorship*

** * Corresponding author: jane.mckeating@ndm.ox.ac.uk*

†*deceased co-author*

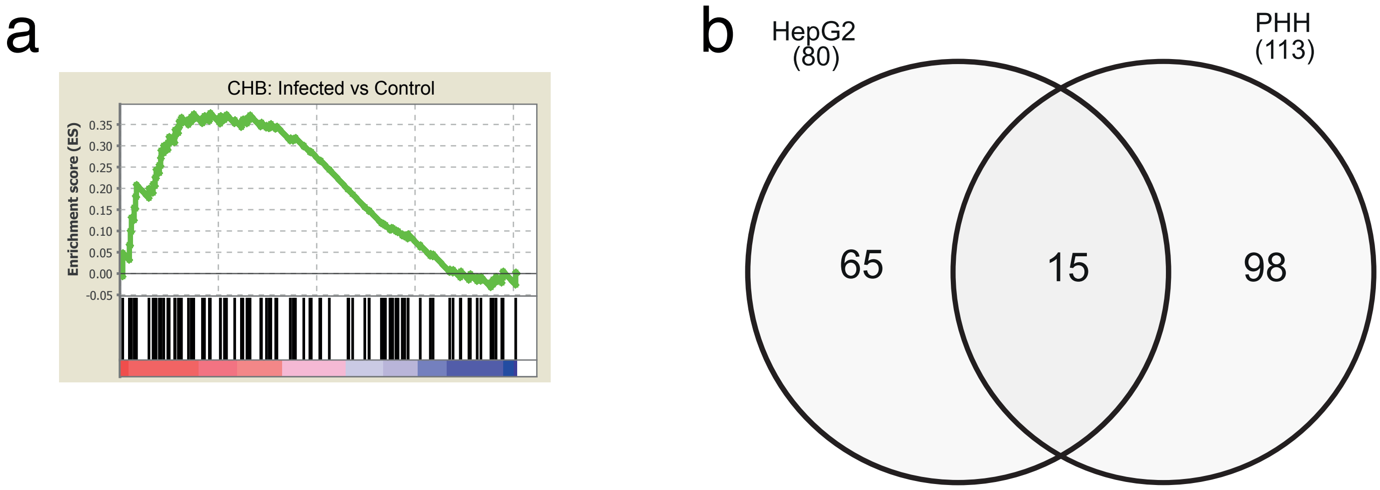

Supplementary figure 1. Increased primary human hepatocyte hypoxic gene expression in CHB. GSEA shows an enrichment of primary human hepatocyte (PHH) derived hypoxic genes in CHB cohort vs healthy controls (FDR=0.351); GSEA was performed using GSEA_4.0.3 (a). Differential gene expression in HepG2 cells (0.5% oxygen for 16h) and PHHs (1% oxygen for 4h)72, using a cut-off of at least 2 fold change, with an FDR of 0.05, showed 80 differentially expressed genes in HepG2 cells and 113 in PHHs, with 15 common overlapping genes (b).

HBx

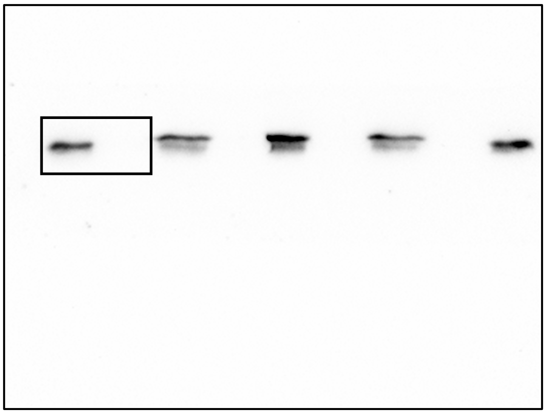

Smc6

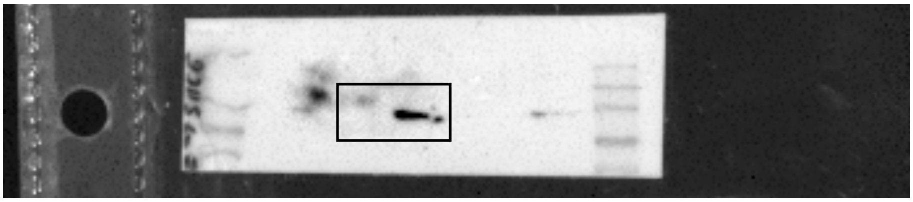

β-actin

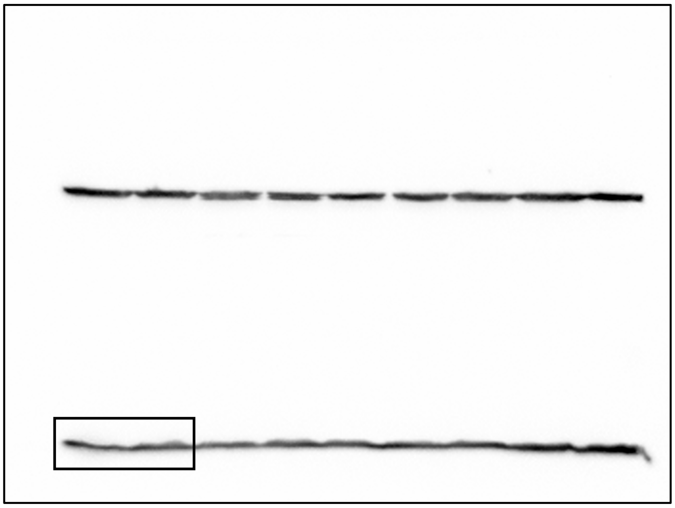

Supplementary figure 2. Uncropped western blots shown for Fig.2a with relevant samples highlighted. HBx and β-actin images originate from the same PVDF membrane cut at the 70kDa molecular marker position. Smc6 image was developed on a separate PVDF membrane.

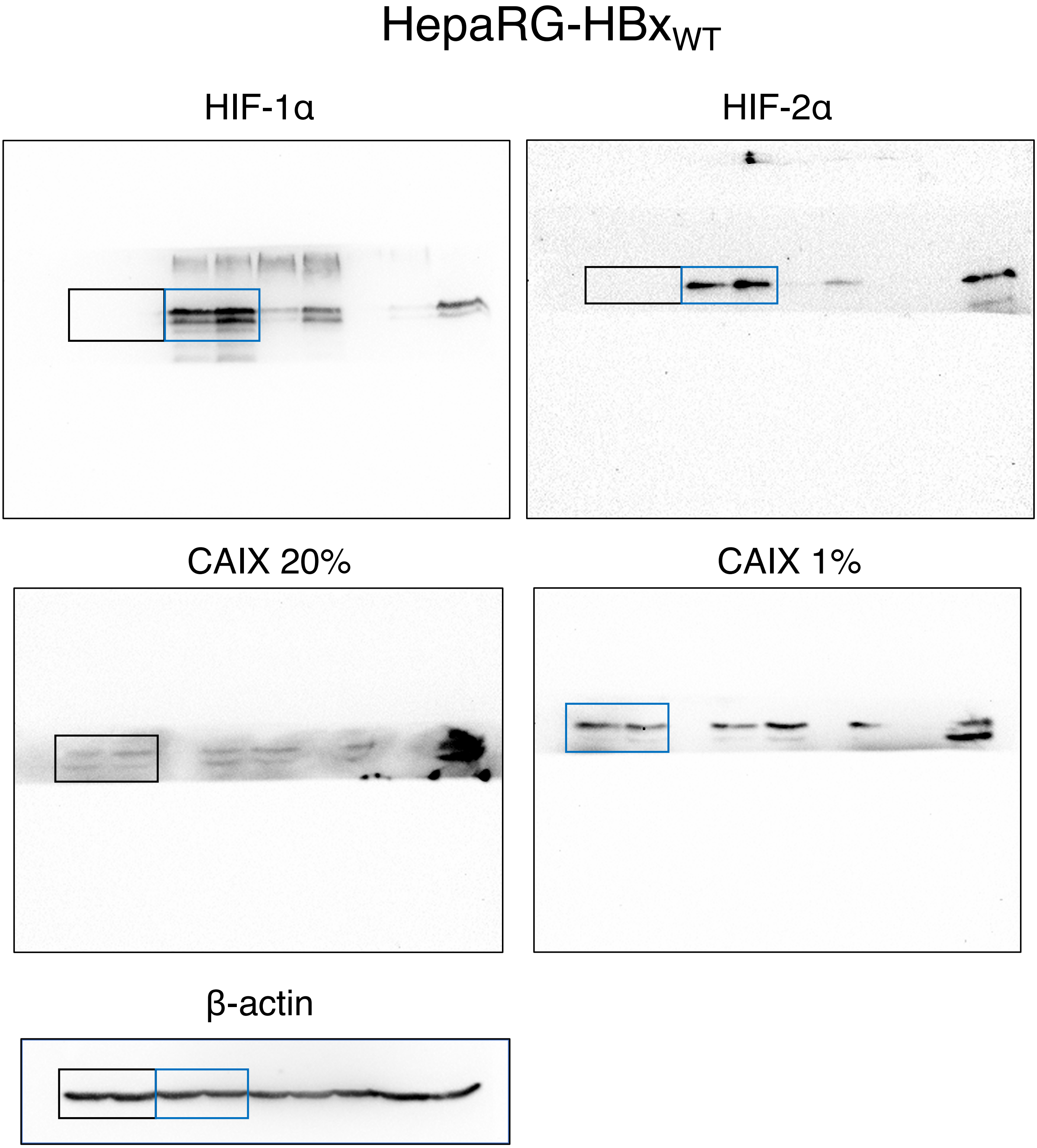

Supplementary figure 3. Uncropped western blots shown for Fig.2c (left panel) HepaRG-HBx$_{WT}$ with relevant samples highlighted (black for 20% and blue for 1% oxygen). HIF-1α, HIF-2α and β-actin images were from the same PVDF membrane cut at the 70kDa molecular marker position. CAIX images were developed on separate PVDF membranes.

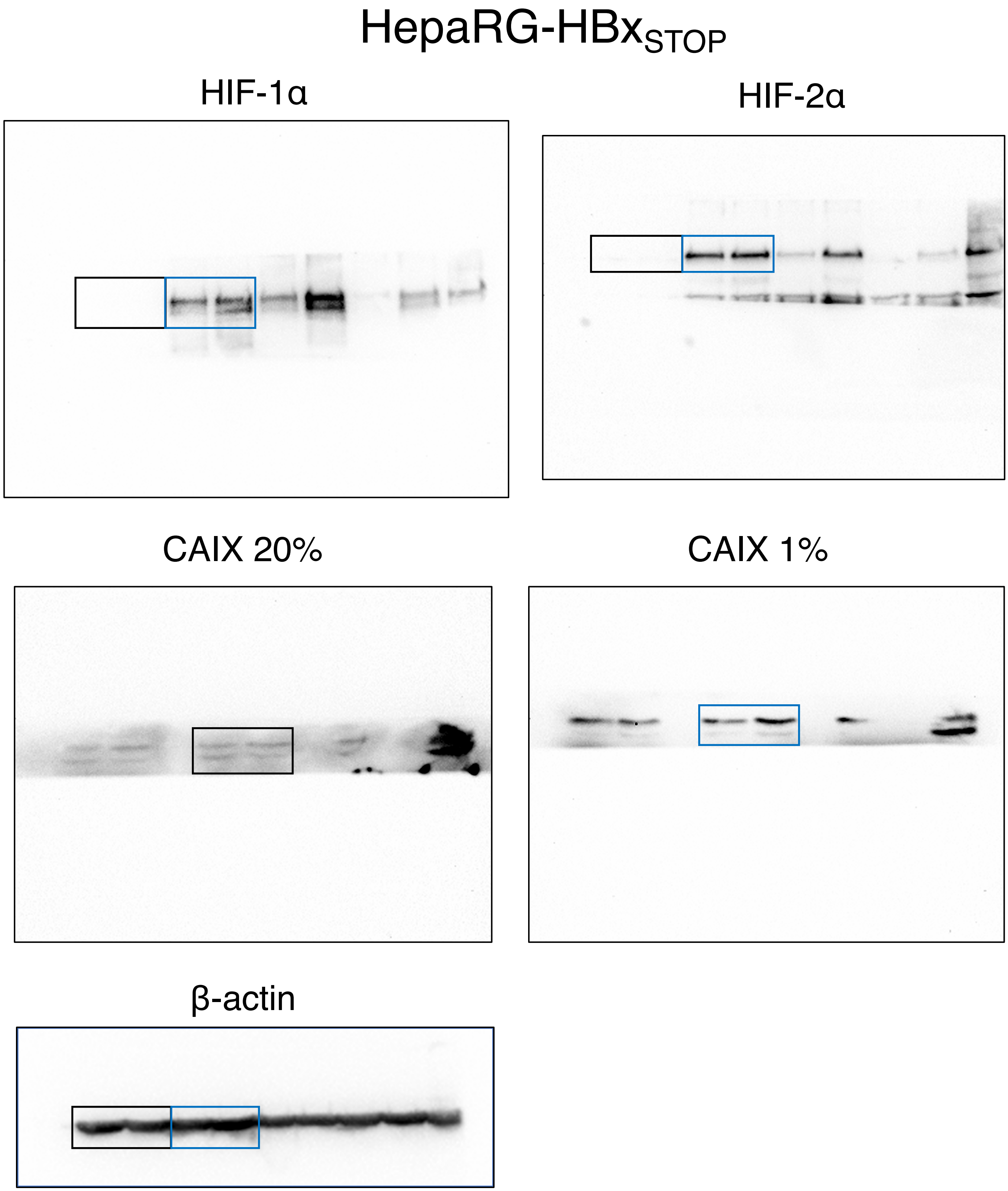

Supplementary figure 4. Uncropped western blots shown for Fig.2c (right panel) HepaRG-HBx$_{STOP}$ with relevant samples highlighted (black for 20% and blue for 1% oxygen). HIF-1α, HIF-2α and β-actin images were from the same PVDF membrane cut at the 70kDa molecular marker position. CAIX images were developed on separate PVDF membranes.

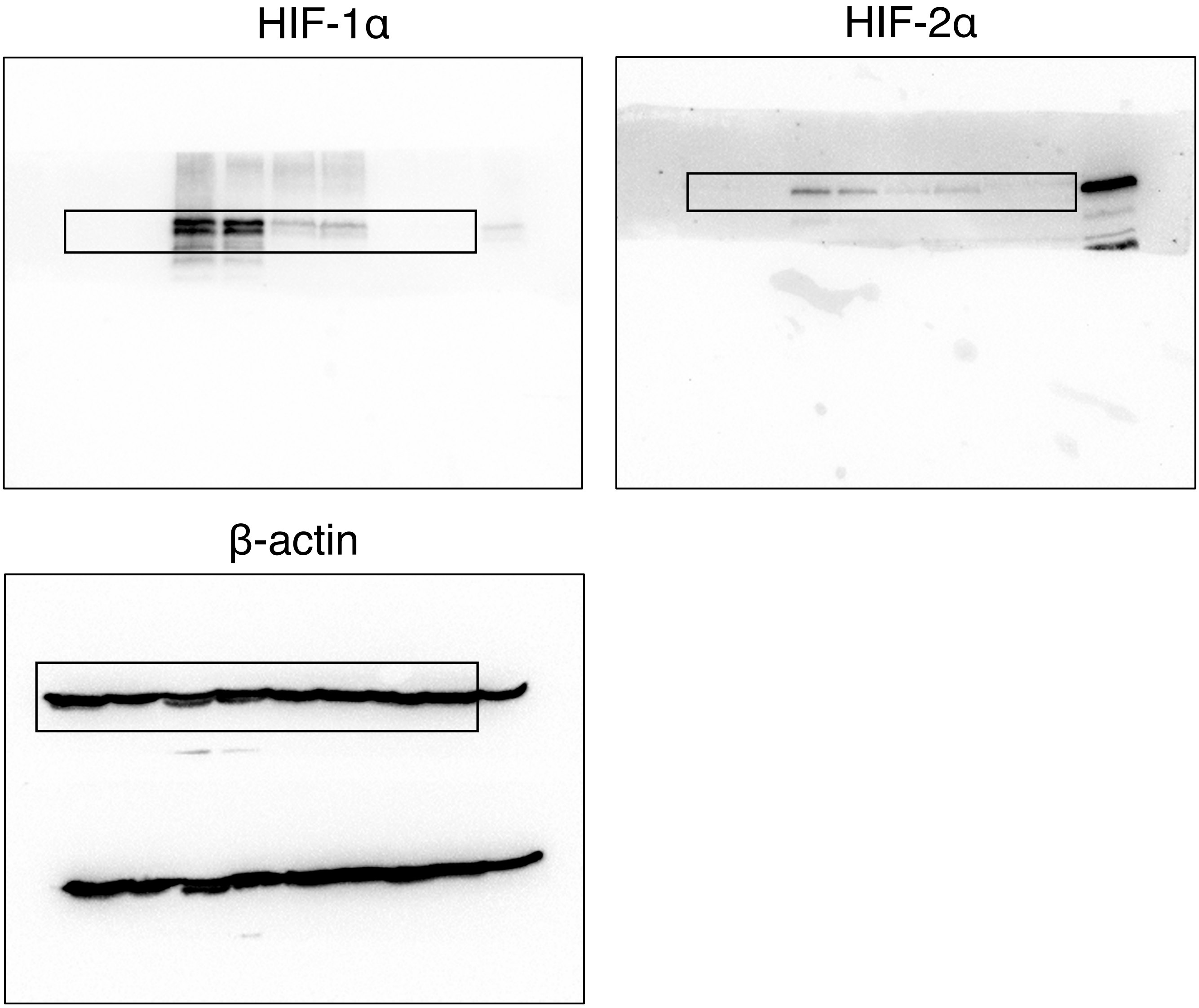

Supplementary figure 5. Uncropped western blots shown for Fig.2e with relevant samples highlighted. HIF-1α, HIF-2α and β-actin images were from the same PVDF membrane cut at the 70kDa molecular marker position.

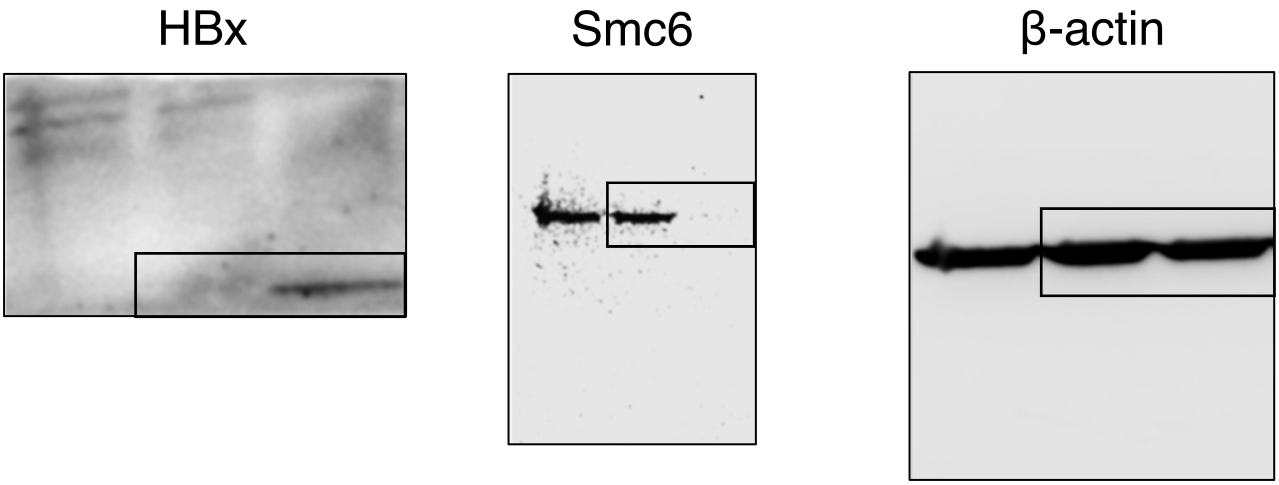

Supplementary figure 6. Uncropped western blots shown for Fig.3a with relevant samples highlighted. Smc6 and β-actin images were from the same PVDF membrane cut at the 70kDa molecular marker position. HBx image was developed on a separate membrane.

Ad-HBx

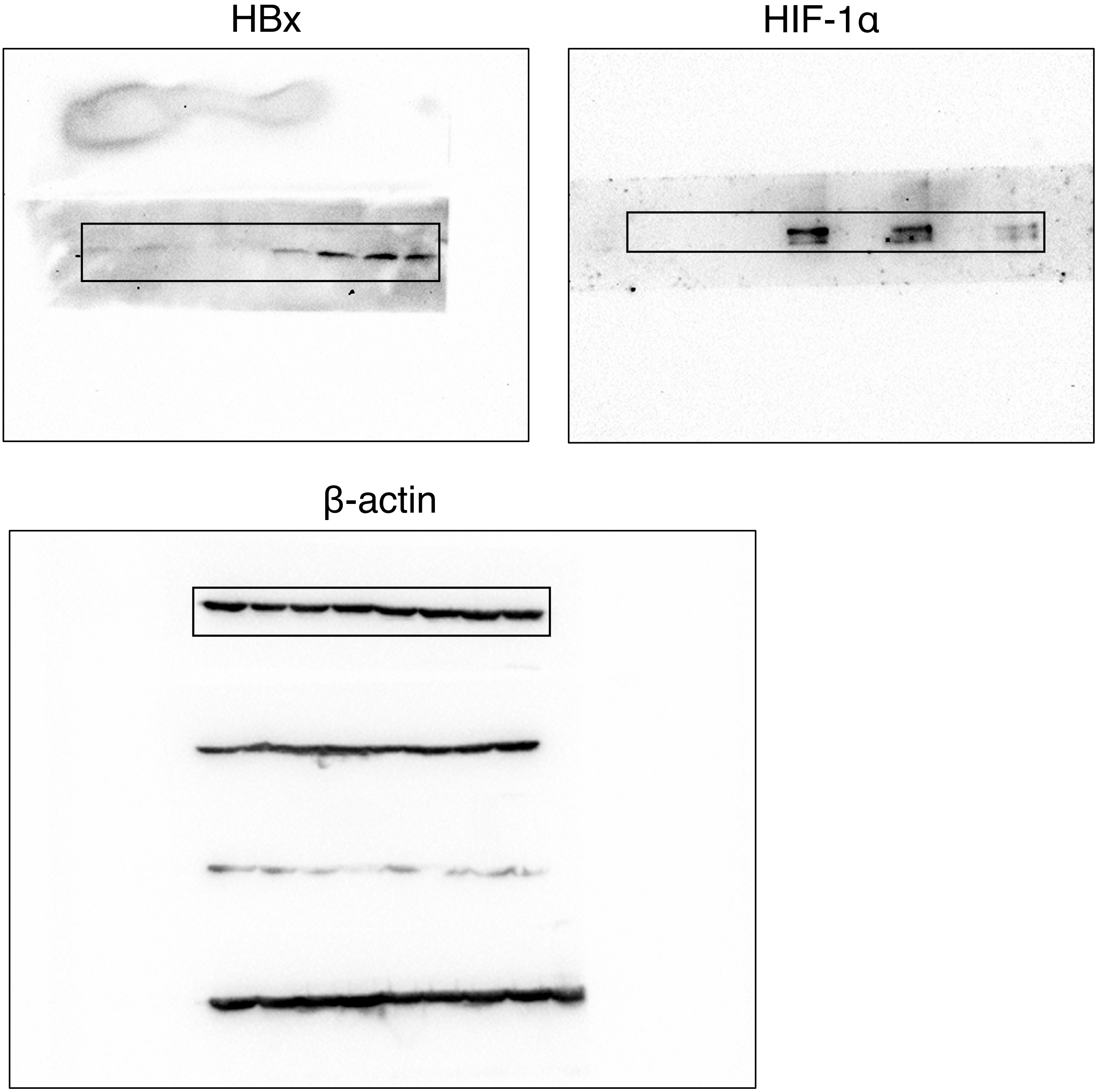

Supplementary figure 7. Uncropped western blots shown for Fig.3b (upper panel) Ad-HBx with relevant samples highlighted. HIF-1α and β-actin images were from the same PVDF membrane cut at the 70kDa molecular marker position. HBx image was developed on a separate membrane

Ad-OVA

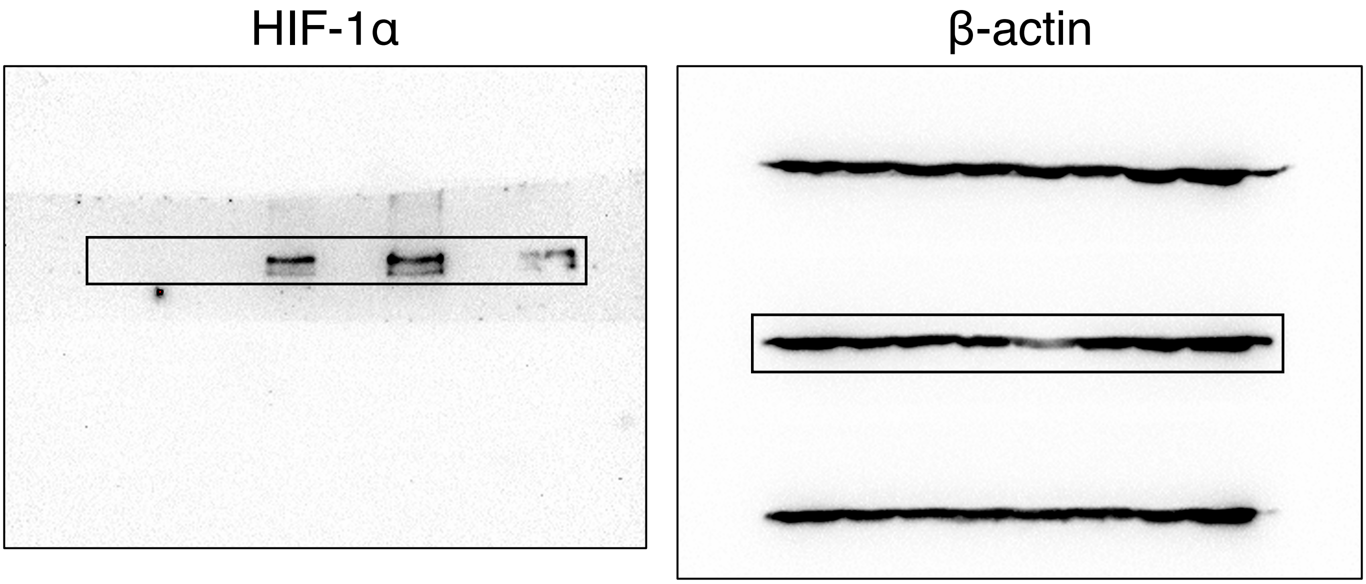

Supplementary figure 8. Uncropped western blots shown for Fig.3b (lower panel) Ad-OVA with relevant samples highlighted. HIF-1α and β-actin images were from the same PVDF membrane cut at the 70kDa molecular marker position.

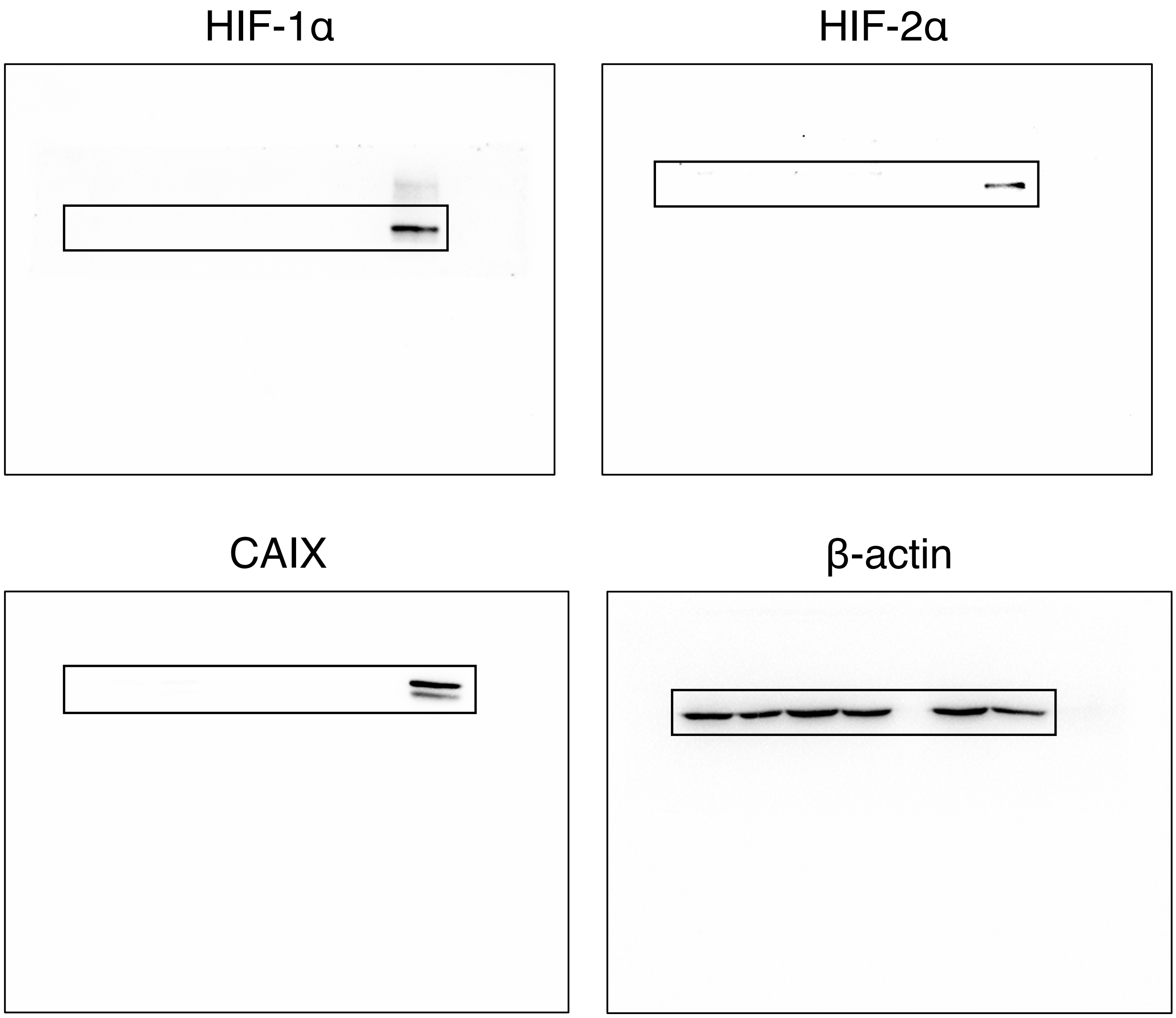

Supplementary figure 9. Uncropped western blots shown for Fig.5a with relevant samples highlighted. HIF-1α/ HIF-2α and CAIX/β-actin images were from the same PVDF membrane cut at the 70kDa molecular marker position.

HYPOXIA_UPREGULATED_GENE_SIGNATURES				
RANK	SIGNATURE	SIZE	OVERLAP	ORIGIN
1	CERVERA SDHB TARGETS 2	111	1	Hep3B and HeLA Cells
2	CERVERA SDHB TARGETS 1 UP	113	2	Hep3B and HeLA Cells
3	HARRIS HYPOXIA	80	12	Pan-cancer
4	KRIEG HYPOXIA VIA KDM3A	50	2	Renal and colon carcinoma
5	KENNY CTNNB1 TARGETS UP	48	1	Breast and colon cancer
6	GROSS HYPOXIA VIA ELK3 UP	197	1	Skin endothelium
7	ELVIDGE HYPOXIA BY DMOG UP	130	26	Breast cancer cell line MCF7
8	GROSS ELK3 TARGETS UP	27	0	Skin endothelium
9	ELVIDGE HYPOXIA UP	164	29	Breast cancer cell line MCF7
10	HU ANGIOGENESIS UP	20	0	nSCLC
11	GROSS HYPOXIA VIA ELK3 ONLY UP	31	2	Skin endothelium
12	WINTER HYPOXIA METAGENE	232	25	Head and neck cancers
13	QI HYPOXIA TARGETS OF HIF1A AND FOXA2	30	3	Adeno- and prostate carcinoma
14	BIOCARTA P53 HYPOXIA PATHWAY	20	1	Cardiovascular endothelium
15	DER IFN ALPHA RESPONSE UP	70	1	Fibroscarcoma
16	MANALO HYPOXIA UP	201	23	Pulmonary artery endothelium
17	WEINMANN ADAPTATION TO HYPOXIA UP	25	0	Hypoxia tolerant lung cancer cells
18	LEONARD HYPOXIA	42	10	Kidney tubule cells
19	KIM HYPOXIA	19	6	Fibroblasts

Supplementary Table 1. Hypoxic gene signatures from MSigDB that are enriched (FDR<0.05) in CHB. Gene signatures are ranked by NES. The table also shows the number of genes in each signature (Size); the number of genes in common with the HepG2 derived signature (Overlap); and the tissue or cell line from which this signature was derived (Origin).

80 Hypoxia signature genes

Rank	Gene_Symbol	Gene_Description	Accession_Number(s)
1	LOXL2	Lysyl oxidase like 2	NM_002318
2	SMIM3	Small integral membrane protein 3	NM_032947
3	LOC154761	Family with sequence similarity 115, member c pseudogene	NR_015421
4	WDR54	Wd repeat domain 54	NM_032118 /// XM_005264586 /// XM_006712111
5	DOK3	Docking protein 3	NM_001144875 /// NM_001144876 /// NM_001308235 /// NM_001308236 /// NM_024872 /// XM_00
6	HCAR3	Hydroxycarboxylic acid receptor 3	NM_006018
7	EHD2	Eh domain containing 2	NM_014601
8	TNS1	Tensin 1	NM_001308022 /// NM_001308023 /// NM_022648 /// XM_011511711 /// XM_011511712 /// XM_01
9	HK1	Hexokinase 1	NM_000188 /// NM_033496 /// NM_033497 /// NM_033498 /// NM_033500 /// XM_005269735 ///
10	SLC2A3	Solute carrier family 2 member 3	NM_006931
11	GYS1	Glycogen synthase 1	NM_001161587 /// NM_002103 /// NR_027763
12	PLAC8	Placenta specific 8	NM_001130715 /// NM_001130716 /// NM_016619
13	IGFBP1	Insulin like growth factor binding protein 1	NM_000596 /// NM_001013029
14	FXYD1	Fxyd domain containing ion transport regulator 1	NM_001278717 /// NM_001278718 /// NM_005031 /// NM_021902
15	LSP1	Lymphocyte-specific protein 1	NM_001013253 /// NM_001013254 /// NM_001013255 /// NM_001242932 /// NM_001289005 /// NM
16	SLC6A8	Solute carrier family 6 member 8	NM_001142805 /// NM_001142806 /// NM_005629
17	TFF1	Trefoil factor 1	NM_003225
18	TUBB1	Tubulin beta 1 class vi	NM_030773
19	RIMKLA	Ribosomal modification protein rimk like family member a	NM_173642 /// XM_006710585
20	PFKFB3	6-phosphofructo-2-kinase/fructose-2,6-biphosphatase 3	NM_001145443 /// NM_001282630 /// NM_001314063 /// NM_004566 /// XM_005252463 /// XM_00
21	CKB	Creatine kinase b	NM_001823
22	RASSF5	Ras association domain family member 5	NM_031437 /// NM_182663 /// NM_182664 /// NM_182665
23	SLC51A	Solute carrier family 51 alpha subunit	NM_152672
24	SPAG4	Sperm associated antigen 4	NM_003116 /// XM_005260519 /// XM_005260520 /// XM_011529009 /// XM_011529010 /// XM_01
25	HAVCR1	Hepatitis a virus cellular receptor 1	NM_001099414 /// NM_001173393 /// NM_001308156 /// NM_012206 /// XM_006714840 /// XM_01
26	ANGPTL4	Angiopoietin like 4	NM_001039667 /// NM_016109 /// NM_139314 /// NR_104213 /// XM_005272484 /// XM_00527248
27	LOX	Lysyl oxidase	NM_001178102 /// NM_001317073 /// NM_002317
28	CA9	Carbonic anhydrase 9	NM_001216 /// XM_006716869 /// XM_006716870 /// XR_428428
29	IGLON5	Iglon family member 5	NM_001101372
30	PFKFB4	6-phosphofructo-2-kinase/fructose-2,6-biphosphatase 4	NM_004567 /// XM_005265230 /// XM_005265231 /// XM_011533829 /// XM_011533830 /// XM_01
31	PLEKHA2	Pleckstrin homology domain containing a2	NM_021623 /// XM_011544605 /// XM_011544606 /// XM_011544607 /// XM_011544608
32	LOXL3	Lysyl oxidase like 3	NM_001289164 /// NM_001289165 /// NM_032603 /// XM_011533134
33	CHST15	Carbohydrate (n-acetylgalactosamine 4-sulfate 6-o) sulfotransferase 15	NM_001270764 /// NM_001270765 /// NM_014863 /// NM_015892 /// XM_005269891 /// XM_00526
34	TMCC1	Transmembrane and coiled-coil domain family 1	NM_001017395 /// NM_001128224 /// NM_015008 /// NR_033361 /// XM_006713542 /// XM_00671
35	BHLHE40	Basic helix-loop-helix family member e40	NM_003670
36	EGFL7	Egf like domain multiple 7	NM_016215 /// NM_201446 /// NR_045110 /// NR_045111 /// NR_046367 /// XM_006717141 ///
37	EGLN3	Egl-9 family hypoxia inducible factor 3	NM_001308103 /// NM_022073 /// XM_006720015
38	KCTD11	Potassium channel tetramerization domain containing 11	NM_001002914
39	FGF11	Fibroblast growth factor 11	NM_001303460 /// NM_004112 /// NR_130156
40	TMEM45A	Transmembrane protein 45a	NM_018004 /// XM_005247569
41	ISM2	Isthmin 2	NM_182509 /// NM_199265 /// NM_199296 /// XM_011536489
42	NDRG1	N-myc downstream regulated 1	NM_001135242 /// NM_001258432 /// NM_001258433 /// NM_006096 /// XM_011516791 /// XM_01
43	SERPINE1	Serpin family e member 1	NM_000602 /// NM_001165413
44	ESPN	Espin	NM_031475 /// XM_005263501 /// XM_011542231 /// XM_011542232 /// XM_011542233 /// XM_01
45	ADM	Adrenomedullin	NM_001124
46	RNASET2	Ribonuclease t2	NM_003730
47	FAM110C	Family with sequence similarity 110 member c	NM_001077710 /// XM_011510372 /// XM_011510373 /// XM_011510374
48	IGFBP3	Insulin like growth factor binding protein 3	NM_000598 /// NM_001013398
49	PDK1	Pyruvate dehydrogenase kinase 1	NM_001278549 /// NM_002610 /// NR_103729 /// NR_103731 /// XM_006712594 /// XM_00671259
50	PDGFB	Platelet derived growth factor subunit b	NM_002608 /// NM_033016
51	UNC93A	Protein unc-93 homolog a	NM_001143947 /// NM_018974 /// XM_011535905 /// XM_011535906 /// XM_011535907 /// XM_01
52	PPP2R2C	Protein phosphatase 2 regulatory subunit bgamma	NM_001206994 /// NM_001206995 /// NM_001206996 /// NM_020416 /// NM_181876 /// XM_00524
53	FAM13A	Family with sequence similarity 13 member a	NM_001015045 /// NM_001265578 /// NM_001265579 /// NM_001265580 /// NM_014883 /// XM_00
54	PLIN2	Perilipin 2	NM_001122 /// NR_038064 /// XM_006716719
55	GBE1	Glycogen banching enzyme	NM_000158
56	LCN15	Lipocalin 15	NM_203347 /// XM_006717105 /// XM_011518672
57	ZNF395	Zinc finger protein 395	NM_018660
58	RAB42	Ras-related protein rab-42	NM_001193532 /// NM_152304
59	PGK1	Phosphoglycerate kinase 1	NM_000291
60	PPP1R3C	Protein phosphatase 2 regulatory subunit 3c	NM_005398
61	BNIP3L	Bcl2/adenovirus e1b 19kda protein-interacting protein 3-like	NM_004331 /// XM_005273617 /// XM_011544630
62	ANKRD37	Ankyrin repeat domain 37	NM_181726 /// XM_005262981
63	ANKZF1	Ankyrin repeat and zinc finger domain containing 1	NM_001042410 /// NM_001282792 /// NM_018089 /// XM_005246663 /// XM_011511392 /// XR_42
64	APOL1	Apolipoprotein l1	NM_001136540 /// NM_001136541 /// NM_003661 /// NM_145343 /// NM_145344 /// XM_00526179
65	DDIT4	Dna damage inducible transcript 4	NM_019058
66	PNPLA7	Patatin-like phospholipase domain-containing protein 7	NM_001098537 /// NM_152286 /// XM_006717102 /// XM_006717104 /// XM_011518664 /// XR_92
67	BNIP3	Bcl2 interacting protein 3	NM_004052
68	RORA	Rar related orphan receptor a	NM_002943 /// NM_134260 /// NM_134261 /// NM_134262 /// XM_005254584 /// XM_011521873 /
69	NDUFA4L2	Ndufa4 mitochondrial complex associated like 2	NM_020142 /// XM_005269033 /// XM_011538573
70	PIGZ	Phophatidylinositol glycan anchor biosynthesis class z	NM_025163 /// XM_006713758 /// XM_011513190 /// XM_011513191 /// XM_011513192
71	ABCA7	Atp binding cassette transporter a7	NM_019112 /// NM_033308 /// XM_006722616 /// XM_006722617 /// XM_006722618 /// XM_01152
72	HR	Protein hairless	NM_005144 /// NM_018411 /// XM_005273569 /// XM_006716367
73	EPO	Erythropoietin	NM_000799
74	TMEM145	Transmembrane protein 145	NM_173633 /// XM_005258781 /// XM_011526791 /// XM_011526792
75	MIR210HG	Mir210 host gene	NR_038262
76	CITED2	Cbp/p300 interacting transactivator with glu/asp rich carboxy-terminal domain 2	NM_001168388 /// NM_001168389 /// NM_006079
77	PLOD2	Procollagen-lysine,2-oxoglutarate 5-dioxygenase 2	NM_000935 /// NM_182943 /// XM_005247535 /// XM_005247536
78	INHA	Inhibin alpha	NM_002191
79	BIRC7	Baculoviral iap repeat containing 7	NM_022161 /// NM_139317
80	ALDOC	Aldolase, fructose-bisphosphate c	NM_005165 /// XM_005257949 /// XM_011524556

Supplementary Table 2. Hypoxia upregulated genes in HepG2 cells.
